# Supplementary material for: Effect of linezolid on platelet count in critically ill patients with thrombocytopenia
Source: PLoS One. 2023 Jun 29;18(6):e0286088. doi: 10.1371/journal.pone.0286088 (PMC10310034; doi:10.1371/journal.pone.0286088)
Supplement: Attachment — Submitted filename: PONE-D-22-25412.docx [file pone.0286088.s002.docx]

PLOS ONE

### Effect of linezolid on platelet count in critically ill patients with thrombocytopenia

--Manuscript Draft--

| Manuscript Number: | PONE-D-22-25412 |
| --- | --- |
| Article Type: | Research Article |
| Full Title: | Effect of linezolid on platelet count in critically ill patients with thrombocytopenia |
| Short Title: | Effect of linezolid on platelet count in critically ill patients with thrombocytopenia |
| Corresponding Author: | Hiroomi Tatsumi  Sapporo Medical University School of Medicine Sappporo, Hokkaido JAPAN |
| Keywords: | linezolid; thrombocytopenia; platelet concentrate transfusion; hematological disease |
| Abstract: | Introduction: Linezolid (LZD) is one of the antibiotics used for methicillin-resistant Staphylococcus aureus . In Japan, the dose of LZD is not generally adjusted by renal function or therapeutic drug monitoring (TDM). Therefore, LZD is readily available for critically ill patients. On the other hand, pancytopenia, especially thrombocytopenia, may be observed as an adverse effect of LZD. We investigated the effect of LZD on platelet count in patients with thrombocytopenia.  Methods: Fifty-five critically ill patients with thrombocytopenia (platelet count < 100  ×10 3 /µL) who received LZD for five days or more during the period from January 2011 to October 2018 were included. Changes in platelet count and frequency of platelet concentrate (PC) transfusion were evaluated retrospectively.  Results: Median duration of LZD administration was 9 days. Platelet count was 47  ×10 3 /µL before LZD administration and increased gradually and significantly. The platelet count on day 15 was 86 ×10 3 /µL, which was significantly higher than that before LZD administration. Thirty-two patients (58.2%) required PC transfusion within 15 days after starting LZD administration. The daily rate of PC transfusion (= PC transfused / 55 patients) was 30.2% on days 1-5, which was higher than the rate of 20.7% on days 6-10 and that of 18.2% on days 11-15. Similar tendencies were observed in non-hematological and hematological disease patients.  Conclusion: Since the improvement of serious illness due to the effectiveness of LZD results in an increase in platelet count, there may be considered administering LZD even in patients with thrombocytopenia when LZD is needed. |
| Order of Authors: | Hiroomi Tatsumi |
|  | Akatsuka Masayuki |
|  | Hiromitsu Kuroda |
|  | Satoshi Kazuma |
|  | Shintaro Suzuki |
|  | Yoshiki Masuda |
| Additional Information: |  |
| Question | Response |
| Financial Disclosure  Enter a financial disclosure statement that describes the sources of funding for the work included in this submission. Review the [submission guidelines](http://journals.plos.org/plosone/s/submission-guidelines#loc-financial-disclosure-statement) for detailed requirements. View published research articles from [*PLOS ONE*](http://journals.plos.org/plosone/) for specific examples. | The funders had no role in study design, data collection and analysis, decision to publish, or preparation of the manuscript. |

| This statement is required for submission and will appear in the published article if the submission is accepted. Please make sure it is accurate. |  |
| --- | --- |
| Unfunded studies  Enter: *The author(s) received no specific funding for this work.*  Funded studies  Enter a statement with the following details:   - Initials of the authors who received each award - Grant numbers awarded to each author - The full name of each funder - URL of each funder website - Did the sponsors or funders play any role in the study design, data collection and analysis, decision to publish, or preparation of the manuscript? - NO - Include this sentence at the end of   your statement: *The funders had no role in study design, data collection and analysis, decision to publish, or preparation of the manuscript.*   - YES - Specify the role(s) played. |  |
| * typeset |  |
| Competing Interests  Use the instructions below to enter a competing interest statement for this submission. On behalf of all authors, disclose any [competing interests](http://journals.plos.org/plosone/s/competing-interests) that could be perceived to bias this work—acknowledging all financial support and any other relevant financial or non­financial competing interests.  This statement is required for submission and will appear in the published article if the submission is accepted. Please make sure it is accurate and that any funding sources listed in your Funding Information later in the submission form are also declared in your Financial Disclosure statement.  View published research articles from  [*PLOS ONE*](http://journals.plos.org/plosone/) for specific examples. | I have read the journal's policy and the authors of this manuscript have the following competing interests: H.T. received lecture fees from TSUMURA & CO. and industry-academia collaborative research grant from Otsuka Pharmaceutical Factory, Inc. Y.M. received lecture fees from MSD K.K., Asahi Kasei Pharma Corp. and Japan Blood Product and industry-academia collaborative research grant from JIMRO Co., Ltd.  Other authors declare that they have no competing interests. |

| NO authors have competing interests  Enter: *The authors have declared that no competing interests exist.*  Authors with competing interests  Enter competing interest details beginning with this statement:  *I have read the journal's policy and the authors of this manuscript have the following competing interests: [insert competing interests here]* |  |
| --- | --- |
| * typeset |  |
| Ethics Statement  Enter an ethics statement for this submission. This statement is required if the study involved:   - Human participants - Human specimens or tissue - Vertebrate animals or cephalopods - Vertebrate embryos or tissues - Field research   Write "N/A" if the submission does not require an ethics statement.  General guidance is provided below. Consult the [submission guidelines](http://journals.plos.org/plosone/s/submission-guidelines#loc-guidelines-for-specific-study-types) for detailed instructions. Make sure that all information entered here is included in the Methods section of the manuscript. | Approval of the research protocol: The study protocol was approved by the Research Ethics Committee of Sapporo Medical University (approval number 302-145).  Informed Consent: Since this study was a retrospective observational study, obtaining informed consent was waived from individual patients. An information disclosure document about this study was created and was made available to the study patients via website of the hospital ICU, guaranteeing the opportunity for study patients to refuse. |

| Format for specific study types  Human Subject Research (involving human participants and/or tissue)   - Give the name of the institutional review board or ethics committee that approved the study - Include the approval number and/or a statement indicating approval of this research - Indicate the form of consent obtained (written/oral) or the reason that consent was not obtained (e.g. the data were analyzed anonymously)   Animal Research (involving vertebrate animals, embryos or tissues)   - Provide the name of the Institutional Animal Care and Use Committee (IACUC) or other relevant ethics board that reviewed the study protocol, and indicate whether they approved this research or granted a formal waiver of ethical approval - Include an approval number if one was obtained - If the study involved *non-human primates*, add *additional details* about animal welfare and steps taken to ameliorate suffering - If anesthesia, euthanasia, or any kind of animal sacrifice is part of the study, include briefly which substances and/or methods were applied   Field Research  Include the following details if this study involves the collection of plant, animal, or other materials from a natural setting:   - Field permit number - Name of the institution or relevant body that granted permission |  |
| --- | --- |
|  |  |
| Data Availability  Authors are required to make all data underlying the findings described fully available, without restriction, and from the time of publication. PLOS allows rare exceptions to address legal and ethical concerns. See the [PLOS Data Policy](http://journals.plos.org/plosone/s/data-availability) and [FAQ](http://journals.plos.org/plosone/s/data-availability#loc-faqs-for-data-policy) for detailed information. | Yes - all data are fully available without restriction |

| A Data Availability Statement describing where the data can be found is required at submission. Your answers to this question constitute the Data Availability Statement and will be published in the article, if accepted. |  |
| --- | --- |
| Important: Stating ‘data available on request from the author’ is not sufficient. If your data are only available upon request, select ‘No’ for the first question and explain your exceptional situation in the text box. |  |
| Do the authors confirm that all data underlying the findings described in their manuscript are fully available without restriction? |  |
| Describe where the data may be found in full sentences. If you are copying our sample text, replace any instances of XXX with the appropriate details.   - If the data are held or will be held in a public repository, include URLs, accession numbers or DOIs. If this information will only be available after acceptance, indicate this by ticking the box below. For example: *All XXX files are available from the XXX database (accession number(s) XXX, XXX.*). - If the data are all contained within the manuscript and/or Supporting Information files, enter the following: *All relevant data are within the manuscript and its Supporting Information files.* - If neither of these applies but you are able to provide details of access elsewhere, with or without limitations, please do so. For example:   *Data cannot be shared publicly because of [XXX]. Data are available from the XXX Institutional Data Access / Ethics Committee (contact via XXX) for researchers who meet the criteria for access to confidential data.*  *The data underlying the results presented in the study are available from (include the name of the third party* | All relevant data are within the manuscript and its Supporting Information files. |

| *and contact information or URL).*   - This text is appropriate if the data are owned by a third party and authors do not have permission to share the data.   * typeset |  |
| --- | --- |
| Additional data availability information: |  |

<https://www.editorialmanager.com/pone/download.aspx?id=31828562&guid=5d385f3f-41e1-45b1-9c28-831e36de5533&scheme=1>Manuscript	[Click here to](https://www.editorialmanager.com/pone/download.aspx?id=31828562&guid=5d385f3f-41e1-45b1-9c28-831e36de5533&scheme=1)

[access/download;Manuscript;renamed_cbb58.docx](https://www.editorialmanager.com/pone/download.aspx?id=31828562&guid=5d385f3f-41e1-45b1-9c28-831e36de5533&scheme=1)

1	Effect of linezolid on platelet count in critically ill patients with thrombocytopenia

2

3 Hiroomi Tatsumi^1¶*^, Masayuki Akatsuka^1¶^, Hiromitsu Kuroda^1^, Satoshi Kazuma^1^, Shintaro Suzuki^1^,
4 Yoshiki Masuda^1^

5

6 **Affiliations:**
7 1. Department of Intensive Care Medicine, Sapporo Medical University School of Medicine, Sapporo,
8 Hokkaido, Japan

9

10 ***Corresponding author:**
11 Hiroomi Tatsumi
12 E-mail: [htatsumi@sapmed.ac.jp](mailto:htatsumi@sapmed.ac.jp)

13

14	^¶^These authors contributed equally to this work.

15

#### 16 Abstract

17 *Introduction:*	Linezolid (LZD) is one of the antibiotics used for methicillin-resistant *Staphylococcus*
18 *aureus*. In Japan, the dose of LZD is not generally adjusted by renal function or therapeutic drug
19 monitoring (TDM). Therefore, LZD is readily available for critically ill patients. On the other hand,
20 pancytopenia, especially thrombocytopenia, may be observed as an adverse effect of LZD. We
21 investigated the effect of LZD on platelet count in patients with thrombocytopenia.
22 *Methods:*	Fifty-five critically ill patients with thrombocytopenia (platelet count < 100 ×10^3^ /µL)
23 who received LZD for five days or more during the period from January 2011 to October 2018 were
24 included. Changes in platelet count and frequency of platelet concentrate (PC) transfusion were
25 evaluated retrospectively.
26 *Results:*	Median duration of LZD administration was 9 days. Platelet count was 47 ×10^3^ /µL before
27 LZD administration and increased gradually and significantly. The platelet count on day 15 was 86
28 ×10^3^ /µL, which was significantly higher than that before LZD administration. Thirty-two patients
29 (58.2%) required PC transfusion within 15 days after starting LZD administration. The daily rate of
30 PC transfusion (= PC transfused / 55 patients) was 30.2% on days 1-5, which was higher than the rate
31 of 20.7% on days 6-10 and that of 18.2% on days 11-15. Similar tendencies were observed in non-
32 hematological and hematological disease patients.
33 *Conclusion:*	Since the improvement of serious illness due to the effectiveness of LZD results in an
34 increase in the platelet count, administering LZD may be considered even in patients with
35 thrombocytopenia when LZD is needed.

36

#### 37 Keywords

38 linezolid, thrombocytopenia, platelet concentrate transfusion, hematological disease

39

#### 40 Abbreviations

41 MRSA:	methicillin-resistant *Staphylococcus aureus*
42 VRE:	vancomycin-resistant enterococci
43 LZD:	linezolid
44 VAN: vancomycin
45 TDM: therapeutic drug monitoring
46 AKI: acute kidney injury
47 CRRT: continuous renal replacement therapy
48 DIC: disseminated intravascular coagulation
49 PC: platelet concentrate
50 IQR: interquartile range
51 APACHE: acute physiology and chronic health evaluation
52 SOFA: sequential organ failure assessment

53

#### 54 Introduction

55 Among Gram-positive bacteria, methicillin-resistant *Staphylococcus aureus* (MRSA) and
56 vancomycin-resistant enterococci (VRE) are crucial pathogens that cause hospital-acquired infection^1^
57 and are related to high rate of hospital mortality, especially in critically ill patients^2^. Linezolid (LZD)
58 is one of the antibiotics used for MRSA and VRE. Among the various anti-MRSA drugs such as
59 vancomycin (VAN), the dose of LZD is not generally adjusted by renal function or therapeutic drug
60 monitoring (TDM) in Japan. Therefore, LZD is readily available for patients with acute kidney injury
61 (AKI) and patients who are receiving continuous renal replacement therapy (CRRT). On the other
62 hand, pancytopenia, especially thrombocytopenia, may be observed as an adverse effect of LZD.
63 Previous studies have shown that the development of LZD-induced thrombocytopenia may be
64 associated with body weight^3-5^, baseline platelet level^4,^ ^6-8^, prolonged duration of therapy^9-10^, renal
65 function^3,^ ^5-6,^ ^11-12^ and plasma LZD concentration^4,^ ^8,^ ^11^. For this reason, an anti-MRSA drug other than
66 LZD may tend to be selected for the patients with thrombocytopenia regardless of other causes
67 including sepsis, disseminated intravascular coagulation (DIC) or myelosuppression. We investigated
68 the effect of LZD on platelet count in patients with thrombocytopenia.

#### 69 Patients and Methods

70 *Study Design*
71 This study was performed retrospectively at the ICU of a university hospital. The study protocol
72 conformed to the ethical guidelines enshrined in the Declaration of Helsinki and was approved by the
73 Research Ethics Committee of Sapporo Medical University (approval number 302-145). Since this
74 study was a retrospective observational study, obtaining informed consent was waived from
75 individual patients. An information disclosure document about this study was created and was made
76 available to the study patients via website of the hospital ICU, guaranteeing the opportunity for study
77 patients to refuse.
78 *Patients and Setting*
79 The subjects were critically ill patients with thrombocytopenia who received LZD for five days or
80 more during the period from January 2011 to October 2018 in our ICU. Thrombocytopenia in this
81 study was defined as a platelet count less than 100 ×10^3^ /µL at the time of LZD administration.
82 Patients who died within 15 days after the start of LZD administration and those younger than 18
83 years were excluded. As a general rule, the daily dose of LZD was 600 mg twice a day, and the timing
84 of discontinuation of LZD administration was decided at a daily ICU conference with attending
85 physicians and ICU staff. Indications for platelet concentrate (PC) transfusion was also decided at the
86 daily ICU conference, according to the consideration not only the platelet count, but also various
87 factors; such as change in platelet count, presence or absence of bleeding, treatment content such as
88 blood purification therapy and the risk of bleeding.
89 Patient characteristics including age and sex, primary disease that caused ICU admission, changes
90 in platelet count from the first day to 15 days after the start of LZD administration, and PC transfusion
91 from the first day to 15 days after the start of LZD administration were evaluated retrospectively.
92 From day 1 to day 15 of the observational period, the daily rate of PC transfusion (= PC transfused /
93 all patients) was calculated and compared in three periods: early period (days 1-5), middle period
94 (days 6-10) and late period (days 11-15).
95 Because the present study was performed retrospectively, daily platelet counts were not determined
96 in all patients, and the original data had many missing values. The missing data were mostly after
97 ICU discharge, especially on the day after PC transfusion, when platelet count was expected to
98 increase. Therefore, we compensated for the missing data by calculating the average of the measured values from the day prior to the missing day and the day thereafter.
99 The patients were divided into two groups: a hematological disease group and a non-hematological
100 disease group. In the hematological disease group, thrombocytopenia or pancytopenia occurred due
101 to chemotherapy or hematopoietic stem cell transplantation for treatment of hematological diseases
102 or malignancies. Changes in platelet count were also analyzed during LZD administration in each
103 group.
104 *Statistical analysis*
105 Categorical variables and continuous variables of patients’ characteristics were represented by
106 proportions (%) and medians [interquartile range, IQR], respectively. Platelet counts were represented
107 by means ± standard error. Serial changes in platelet counts were assessed using Friedman’s test.
108 When a statistically significant difference was obtained after Friedman’s test, Wilcoxon’s t-test as a
109 post hoc test with Bonferroni correction was performed. Probability values less than 0.05 in a two-
110 sided test were considered statistically significant.

#### 111 Results

112 *Patients’ characteristics*
113 Patients’ characteristics are shown in Table 1. Fifty-five patients were included (Fig. 1). The median
114 age of patients was 69 [62.5-75.5] years and 39 (70.9%) of the 55 patients were male. As indicators
115 of the severity of illness in the patients, acute physiology and chronic health evaluation (APACHE)
116 II score and sequential organ failure assessment (SOFA) score were 24 [20.5-28] and 8 [7-10.5],
117 respectively. Of the 55 patients, 34 (61.8%) were diagnosed as having infections on ICU
118 admission and 26 patients (47.3%) developed disseminated intravascular coagulation (DIC) on ICU
119 admission. In many cases, the effects of chemotherapy and hematopoietic stem cell transplantation
120 were taken into consideration in addition to those considered to be sepsis alone. Among primary
121 diseases, cardiovascular diseases were most common (27.3%) followed by hematological diseases
122 (25.4%) and gastrointestinal diseases (16.4%). Thirty-one patients (56.4%) had AKI on ICU
123 admission, and 42 patients (76.4%) needed CRRT while in the ICU. The median duration of LZD administration was 9 [8-12] days. The median duration of ICU stay was 15 [8.5-23.5] days.

| 127 |  |  | | | |
| --- | --- | --- | --- | --- | --- |
| 128 | **Table 1 Patients’ characteristics** |  |  |  |  |
|  |  |  | All  (n = 55) | Non-hematological  (n = 41) | Hematological  (n = 14) |
|  | Age (year) Gender  Female | 69  16 | [62.5-75.5]  (29.1) | 73 [66-76]  12 (29.3) | 50 [61.8-75.5]  4 (28.5) |
|  | Male | 39 | (70.9) | 29 (70.7) | 10 (71.5) |

Primary disease

| Non-hematological | 41 | (74.6) |  |  |  | |
| --- | --- | --- | --- | --- | --- | --- |
| Cardiovascular |  |  | 15 | (27.3) |  |  |
| Gastrointestinal |  |  | 9 | (16.4) |  |  |
| Hepatobiliary-Pancreatic |  |  | 6 | (10.9) |  |  |
| Respiratory |  |  | 3 | (5.5) |  |  |
| Head and Neck |  |  | 2 | (3.6) |  |  |
| Orthopedic |  |  | 2 | (3.6) |  |  |
| Others |  |  | 4 | (7.3) |  |  |
| Hematological | 14 | (25.4) |  |  |  |  |
| Acute myeloid leukemia |  |  |  |  | 6 | (10.9) |
| Acute lymphoblastic leukemia |  |  |  |  | 4 | (7.3) |
| Myelodysplastic syndromes |  |  |  |  | 2 | (3.6) |
| Others |  |  |  |  | 2 | (3.6) |
| APACHE II | 24 | [20.5-28] | 25 | [19-28] | 23.5 | [21-29.5] |
| SOFA score at ICU admission | 8 | [7-10.5] | 8 | [6-10] | 9 | [8-10.8] |

Presumed cause of thrombocytopenia

| Sepsis | 26 | (47.3) | 26 | (63.4) | 0 |  |
| --- | --- | --- | --- | --- | --- | --- |
| Chemotherapy | 2 | (3.6) | 1 | (2.4) | 1 | (7.1) |
| Chemotherapy + Sepsis | 11 | (20.0) | 7 | (17.1) | 4 | (28.6) |
| Hepatic failure | 1 | (1.8) | 1 | (2.4) | 0 |  |
| Hepatic failure + Sepsis | 4 | (7.3) | 4 | (9.8) | 0 |  |
| Hematopoietic stem cell transplantation | 4 | (7.3) | 0 |  | 4 | (28.6) |
| Hematopoietic stem cell transplantation + Sepsis | 4 | (7.3) | 0 |  | 4 | (28.6) |
| Others | 3 | (5.5) | 2 | (4.9) | 1 | (7.1) |

| Infection on ICU admission (n) | 34 | (61.8) | 25 | (61.0) | 9 | (64.3) |
| --- | --- | --- | --- | --- | --- | --- |
| DIC on ICU admission (n) | 26 | (47.3) | 19 | (46.3) | 7 | (50.0) |
| AKI on ICU admission (n) | 31 | (56.4) | 23 | (56.1) | 8 | (57.1) |

|  | Duration of LZD administration (day) | 9 | [8-12] | 9 | [8-12] | 10 | [7.3-12.8] |
| --- | --- | --- | --- | --- | --- | --- | --- |
|  | Administration of CRRT (n) | 42 | (76.4) | 32 | (78.0) | 10 | (71.4) |
|  | Administration of PC transfusion (n) | 27 | (49.1) | 14 | (34.1) | 13 | (92.9) |
|  | Duration of ICU stay (day) | 15 | [8.5-23.5] | 15 | [8-22] | 16.5 | [9.3-23.8] |
|  | ICU mortality (n) | 13 | (23.6) | 8 | (19.5) | 5 | (35.7) |
|  | 28-day mortality (n) | 16 | (29.1) | 9 | (22.0) | 7 | (50.0) |
| 129 |  |  |  |  |  |  |  |

130 *Changes in platelet count and PC transfusion in all patients*
131 Original changes and corrected changes in platelet count are shown in Fig. 2A and 2B,
132 respectively. Platelet count was 47 ± 4 ×10^3^ /µL before LZD administration and increased gradually
133 and significantly (p < 0.01 in original and corrected data). The platelet counts on day 15 were 86 ±
134 13 ×10^3^ /µL in original data and 95 ± 13 ×10^3^ /µL in corrected data, which were significantly higher
135 than that before LZD administration (p < 0.01 in original and corrected data). Thirty-two patients
136 (58.2%) required PC transfusion within 15 days after starting LZD administration. The daily rate of
137 PC transfusion (= PC transfused / 55 patients) after starting LZD administration was 30.2% in early
138 period (day 1-5), which was higher than 20.7% in middle period (day 6-10) and 18.2% in late period
139 (day 11-15).
140 *Changes in platelet count and PC transfusion in non-hematological disease patients*
141 Sub-analyses in 41 patients whose primary diseases were other than hematological diseases were
142 shown in Fig. 3A and 3B, respectively. A similar tendency was observed even when hematological
143 diseases were excluded from the primary diseases. While platelet count was 56 ± 4 ×10^3^ /µL before
144 LZD administration in the non-hematological disease group, the platelet count increased gradually
145 over 15 days (p < 0.01 in the original and the corrected data). The platelet counts on day 15 were 94
146 ± 16 ×10^3^ /µL in the original data and 100 ± 15 ×10^3^ /µL in the corrected data, which were
147 significantly higher than that before LZD administration (p = 0.02 and < 0.01, respectively).
148 Seventeen patients (41.5%) needed PC transfusion within 15 days after starting LZD administration.
149 The daily rate of PC transfusion after starting LZD administration was 17.1% in the early period,
150 which was higher than 8.8% in the middle period and 7.3% in the late period.
151 *Changes in platelet count and PC transfusion in hematological disease patients*
152 Sub-analysis in 14 patients whose primary diseases were hematological diseases were shown in
153 Fig. 4A and 4B, respectively. A similar tendency was observed even if the primary diseases were
154 limited to hematological diseases. While platelet count was 20 ± 2 ×10^3^ /µL before LZD
155 administration in the hematological disease group, the platelet count increased gradually over 15 days
156 (p < 0.01 in the original and the corrected data). The platelet counts on day 15 was 66 ± 24 ×10^3^ /µL
157 in the original data. The platelet counts on day 15 was 80 ± 26 ×10^3^ /µL in the corrected data, which
158 was significantly higher than that before LZD administration (p = 0.04). Thirteen patients (92.9%)
159 needed PC transfusion within 15 days after starting LZD administration. The daily rate of PC
160 transfusion after starting LZD administration was 68.6% in the early period, which was higher than
161 55.7% in the middle period and 50.0% in the late period.

#### 162 Discussion

163 Several reports^9-10,^ ^13-14^ have shown that long term administration of LZD is associated with
164 thrombocytopenia, however, the effect of administration of LZD on platelet count in patients with
165 thrombocytopenia remains to be elucidated. Our study showed that the platelet count increased
166 gradually and the need for PC transfusion tended to decrease after LZD administration in patients
167 with platelet counts <100 ×10^3^ /µL at early LZD administration. Moreover, this result
168 showed a similar tendency regardless of whether the primary pathology was hematological or not. This suggests that short term LZD administration does not prohibit recovery in the platelet count among patients with thrombocytopenia due to infections or hematological disorders. In
169 addition, no leukopenia or erythrocytopenia that could be attributed to LZD was observed in this
170 study.
171 It is well known that AKI is more likely to occur in critically ill patients; in this study, more than half of patients had AKI on ICU admission, and 76% of patients needed CRRT while in the ICU.. Since anti-MRSA drugs
172 are generally excreted by the kidneys, the dose of VAN or teicoplanin, which requires TDM, depends on
173 the renal function. When AKI worsens enough to require CRRT, the dosage decisions become
174 more complicated. LZD has a wide safe range of blood concentration, and the dose of LZD is not
175 generally adjusted according to renal function or TDM in Japan, even in such AKI patients or during CRRT.
176 Therefore, LZD is easy to use as anti-MRSA drug for the treatment of critically ill patients.
15. Moreover, LZD has excellent tissue migration properties due to it's low
177 molecular weight (337.35 Da), low protein binding rate (31%), large distribution volume (40-50 L/kg)
178 and lipophilicity. Despite these beneficial features, prescribers may be hesitant to administer LZD in patients with
179 thrombocytopenia, especially those with hematological diseases. Since, the present findings
180 showed that the platelet count improved over 2 weeks of LZD administration (with PC transfusion support as needed), empiric therapy with LZD would appear to be acceptable in patients with thrombocytopenia, particularly if LZD is discontinued in a short period of time after the causative bacteria is confirmed.
181 In general, LZD-induced thrombocytopenia occurs 7 days or more after
182 the start of administration and resolves a few days after discontinuation ^2,^ ^6,^ ^13-14,^ ^17-20^. In the
183 present study, it was considered that the platelet count increased as a result of effective
184 treatments that included LZD administration in critically ill patients with thrombocytopenia. The improvement of serious illness due to the efficacy of LZD may occur earlier than LZD-induced thrombocytopenia in many cases. In fact, in this study, the median LZD duration was relatively short (9 days). It is likely that LZD-induced myelosuppression did not occur because the patients recovered earlier than the time when LZD-induced thrombocytopenia might be expected to occur . In other words, if early administration of LZD is effective and LZD
185 administration is discontinued after a short period, thrombocytopenia due to bone marrow suppression
186 caused by LZD may be avoidable. Even in critically ill patients with thrombocytopenia, LZD
187 administration may be considered when needed for the improvement of serious
188 illness due to MRSA infection.
189 The present study has several limitations. Firstly, this study was retrospective, single arm,
190 observational study in a single institute. Unfortunately, the
191 number of patients with thrombocytopenia who were receiving anti-MRSA drugs other than LZD was too
192 small for meaningful comparison between these two groups. Secondly, several platelet count datapoints were missing, which was compensated for by averaging the measured values from the previous and subsequent days. Thirdly, analysis by the causes of thrombocytopenia before LZD administration
193 was not performed, because several causes such as infections or anti-cancer drugs occurred
194 simultaneously and could not be discriminated clearly. Fourthly, it cannot be denied that PC
195 transfusion may have affected the increase in platelet count. However, since the frequency of PC transfusions gradually
196 decreased over the course of treatment, it was considered that the platelet count
197 was recovering despite the administration of LZD due to recovery of the patients general condition. Finally, there was no clear criteria for LZD continuation or discontinuation and PC
198 transfusion; these definitions were determined by reviewing the clinical necessity at daily ICU
199 conference with the attending physicians and ICU staff.

#### 200 Conclusion

201 We evaluated the effect of LZD administration on platelet count in critically ill patients with thrombocytopenia.
202 Since LZD resulted in improvement of serious illness due to MRSA infection with a gradual improvement in the platelet
203 count, LZD administration can be considered (together with PC transfusion as required) in patients with thrombocytopenia if LZD is
204 appropriate for MRSA treatment. When administering LZD to patients with thrombocytopenia, it is
205 desirable to pay attention to changes in the platelet count and to discontinue LZD promptly after
206 improvements of disease and general condition. In the future, a prospective and multicenter study is
207 needed to confirm the safety of LZD use in patients with thrombocytopenia.

236

#### 237 Acknowledgements

238

239 **Approval of the research protocol:** The study protocol was approved by the Research Ethics
240 Committee of Sapporo Medical University (approval number 302-145).
241 **Informed Consent:** Since this study was a retrospective observational study, obtaining informed
242 consent was waived from individual patients. An information disclosure document about this study
243 was created and was made available to the study patients via website of the hospital ICU, guaranteeing
244 the opportunity for study patients to refuse.

#### 245 Registry and the Registration No. of the study/Trial: N/A

246 **Animal Studies:** N/A
247 **Conflict of Interest:** H.T. received lecture fees from TSUMURA & CO. and industry-academia
248 collaborative research grant from Otsuka Pharmaceutical Factory, Inc. Y.M. received lecture fees
249 from MSD K.K., Asahi Kasei Pharma Corp. and Japan Blood Product and industry-academia
250 collaborative research grant from JIMRO Co., Ltd. Other authors declare that they have no competing
251 interests.
252 **Funding Sources:** This research did not receive any specific grant from funding agencies in the
253 public, commercial, or not-for-profit sectors.
254 **ICMJE Statement:** Contributors H.T. conceived of the study, carried out the data acquisition and
255 database construction. M.A., H.K. and S.K. contributed for data analysis and to draft the manuscript.
256 S.S. helped database construction. Y.M. participated in the design of the study, checked the statistical
257 analysis and helped to draft the manuscript. All authors contributed to the writing of the final
258 manuscript.

259

#### 260 References

261 1. Backx M, Healy B. Serious staphylococcal infections. Clin Med. 2008;8:535-8.
262 2. Zhang Z, Liang Z, Li H, Chen L, She D. Comparative evaluation of thrombocytopenia in adult
263 patients receiving linezolid or glycopeptides in a respiratory intensive care unit. Exp Ther Med.
264 2014;7:501-7.
265 3. Natsumoto B, Yokota K, Omata F, Furukawa K. Risk factors for linezolid-associated
266 thrombocytopenia in adult patients. Infection. 2014;42:1007-12.
267 4. Dong HY, Xie J, Chen LH, Wang TT, Zhao YR, Dong YL. Therapeutic drug monitoring and
268 receiver operating characteristic curve prediction may reduce the development of linezolid-associated
269 thrombocytopenia in critically ill patients. Eur J Clin Microbiol Infect Dis. 2014;33:1029-35.
270 5. Tsuji Y, Holford NHG, Kasai H, Ogami C, Heo YA, Higashi Y, et al. Population pharmacokinetics
271 and pharmacodynamics of linezolid-induced thrombocytopenia in hospitalized patients. Br J Clin
272 Pharmacol. 2017;83:1758-72.
273 6. Kaya Kılıç E, Bulut C, Sönmezer MÇ, Ozel Ö, Ataman Hatipoğlu Ç, Tuncer Ertem G, et al. Risk
274 factors for linezolid-associated thrombocytopenia and negative effect of carbapenem combination. J
275 Infect Dev Ctries. 2019;13:886-91.
276 7. Choi GW, Lee JY, Chang MJ, Kim YK, Cho Y, Yu YM, et al. Risk factors for linezolid‐induced
277 thrombocytopenia in patients without haemato-oncologic diseases. Basic Clin Pharmacol Toxicol.
278 2019;124:228-34.
279 8. Cazavet J, Bounes FV, Ruiz S, Seguin T, Crognier L, Rouget A, et al. Risk factor analysis for
280 linezolid-associated thrombocytopenia in critically ill patients. Eur J Clin Microbiol Infect Dis.
281 2020;39:527-38.
282 9. Hirano R, Sakamoto Y, Tachibana N, Ohnishi M. Retrospective analysis of the risk factors for
283 linezolid-induced thrombocytopenia in adult Japanese patients. Int J Clin Pharm. 2014;36:795-9.
284 10. Ichie T, Suzuki D, Yasui K, Takahashi H, Matsuda M, Hayashi H, et al. The association between
285 risk factors and time of onset for thrombocytopenia in Japanese patients receiving linezolid therapy:
286 a retrospective analysis. J Clin Pharm Ther. 2015;40:279-84.
287 11. Nukui Y, Hatakeyama S, Okamoto K, Yamamoto T, Hisaka A, Suzuki H, et al. High plasma
288 linezolid concentration and impaired renal function affect development of linezolid-induced
289 thrombocytopenia. J Antimicrob Chemother. 2013;68:2128-33.
290 12. Kim HS, Lee E, Cho YJ, Lee YJ, Rhie SJ. Linezolid-induced thrombocytopenia increases
291 mortality risk in intensive care unit patients, a 10 year retrospective study. J Clin Pharm Ther.
292 2019;44:84-90.
293 13. Attassi K, Hershberger E, Alam R, Zervos MJ. Thrombocytopenia associated with linezolid
294 therapy. Clin Infect Dis 2002;34:695-8.
295 14. Takahashi Y, Takesue Y, Nakajima K, Ichiki K, Tsuchida T, Tatsumi S, et al. Risk factors
296 associated with the development of thrombocytopenia in patients who received linezolid therapy. J
297 Infect Chemother 2011;17:382-7.
298 15. Crass RL, Cojutti PG, Pai MP, Pea F. Reappraisal of linezolid dosing in renal impairment to
299 improve safety. Antimicrob Agents Chemother 2019;63:e00605-19.
300 16. Rao GG, Konicki R, Cattaneo D, Alffenaar JW, Marriott DJE, Neely M , IATDMCT
301 Antimicrobial Scientific Committee. Therapeutic drug monitoring can improve linezolid dosing
302 regimens in current clinical practice: A review of linezolid pharmacokinetics and pharmacodynamics.
303 Ther Drug Monit. 2020;42:83-92.
304 17. Orrick JJ, Johns T, Janelle J, Ramphal R. Thrombocytopenia secondary to linezolid
305 administration: what is the risk? Clin Infect Dis 2002;35:348‑ 9.
306 18. Rubinstein E, Isturiz R, Standiford HC, Smith LG, Oliphant TH, Cammarata S, et al. Worldwide
307 assessment of linezolid's clinical safety and tolerability: comparator‑ controlled phase III studies.
308 Antimicrob Agents Chemother 2003;47:1824‑ 31.
309 19. Bi LQ, Zhou J, Huang M, Zhou SM. Efficacy of linezolid on gram-positive bacterial infection in
310 elderly patients and the risk factors associated with thrombocytopenia. Pak J Med Sci. 2013;29:837-
311 42.
312 20. Hanai Y, Matsuo K, Ogawa M, Higashi A, Kimura I, Hirayama S, et al. A retrospective study of
313 the risk factors for linezolid-induced thrombocytopenia and anemia. J Infect Chemother.
314 2016;22:536-42.

315

#### 316 Figure legends

317 Fig. 1	Flow chart of patients’ inclusion criteria
318 Fig. 2	Changes in platelet count in all patients
319 A. Original data. Platelet count increased gradually and significantly (p < 0.01). The
320 platelet counts on day 15 was significantly higher than that before LZD administration (86 ± 13 ×10^3^
321 /µL and 47 ± 4 ×10^3^ /µL, respectively; p < 0.01). There were missing values after the 9th day.
322 B. Corrected data. Platelet count increased gradually and significantly (p < 0.01). The
323 platelet counts on day 15 was significantly higher than that before LZD administration (95 ± 13 ×10^3^
324 /µL and 47 ± 4 ×10^3^ /µL, respectively; p < 0.01).
325 Fig. 3	Changes in platelet count in non-hematological disease patients
326 A. Original data. Platelet count increased gradually and significantly (p < 0.01). The
327 platelet counts on day 15 was significantly higher than that before LZD administration (94 ± 16 ×10^3^
328 /µL and 56 ± 4 ×10^3^ /µL, respectively; p = 0.02). There were missing values after the 9th day.
329 B. Corrected data. Platelet count increased gradually and significantly (p < 0.01). The
330 platelet counts on day 15 was significantly higher than that before LZD administration (100 ± 15 ×10^3^
331 /µL and 56 ± 4 ×10^3^ /µL, respectively; p ＜0.01).
332 Fig. 4	Changes in platelet count in hematological disease patients
333 A. Original data. Platelet count increased gradually and significantly (p < 0.01). The
334 platelet counts on day 15 tended to be higher than that before LZD administration (66 ± 24 ×10^3^ /µL
335 and 20 ± 2 ×10^3^ /µL, respectively; p = 0.08). There were missing values after the 14th day.
336 B. Corrected data. Platelet count increased gradually and significantly (p < 0.01). The
337 platelet counts on day 15 was significantly higher than that before LZD administration (80 ± 26 ×10^3^
338 /µL and 20 ± 2 ×10^3^ /µL, respectively; p = 0.04).
339 The platelet counts on day 15 was 66 ± 24 ×10^3^ /µL in the original data. The platelet counts on day
340 15 was 80 ± 26 ×10^3^ /µL in the corrected data, which was significantly higher than that before LZD
341 administration (p = 0.040).

342

<https://www.editorialmanager.com/pone/download.aspx?id=31827960&guid=c1ad7628-1789-4f16-bc77-99d6c5b72324&scheme=1>FFigiugre. 1

| All ICU patients from 2011.1-2018.10  n = 4849 | |  | |
| --- | --- | --- | --- |
|  |  |  | Patients not administered anti-MRSA drugs n = 4432 |
|  |  | |  |
|  |  | |  |
| All ICU patients from 2011.1-2018.10  n = 417 | |  |  |
|  |  |  | |
|  |  |  | Patients not administered LZD n = 161 |
|  |  | |  |
|  |  | |  |
| Patients administered LZD  n = 256 | |  |  |
|  |  |  | |
|  |  |  | Patients administered LZD < 5 days n = 116 |
|  |  | |  |
|  |  | |  |
| Patients administered LZD ≥  5 days  n = 140 | |  |  |
|  |  |  | |
|  |  |  | Patients without thrombocytopenia n = 72 |
|  |  | |  |
|  |  | |  |
| Patients with  thrombocytopenia n = 68 | |  |  |
|  |  |  | |
|  |  |  | Excluded patients：  Died within 15 days after LZD administration (n = 11)  < 18 years (n = 2) |
|  |  | |  |
|  |  | |  |
| Patients including  in this study n = 55 | |  |  |
|  |  |  | |

[Click here to access/download;Figure;renamed_4766d.pptx](https://www.editorialmanager.com/pone/download.aspx?id=31827960&guid=c1ad7628-1789-4f16-bc77-99d6c5b72324&scheme=1)

Fig. 2

# A B

×10^3^ /µL

10

8

12

## Changes in platelet count

### (n = 55)

×10^3^ /µL

10

8

12

### Corrected changes in platelet count

(n = 55)

day	day

6

4

2

0

6

4

2

0

120

100

80

60

40

20

0

1	2	3	4	5	6	7	8	9	10 11 12 13 14 15

120

100

80

60

40

20

0

1	2	3	4	5	6	7	8	9	10 11 12 13 14 15

The number of missing value

Missing rate (%)

1

1.8

1

1.8

1

1.8

1

1.8

1

1.8

14

25.5

7

12.7

Fig. 3

# A B

×10^3^ /µL

10

8

12

10

8

12

120

100

80

60

40

20

0

## Changes in platelet count

### (n = 41)

×10^3^ /µL

## Corrected changes in platelet count

### (n = 41)

1	2	3	4	5	6	7	8	9	10 11 12 13 14 15 day

6

4

2

0

6

4

2

0

120

100

80

60

40

20

0

1	2	3	4	5	6	7	8	9	10 11 12 13 14 15

day

The number of missing value

Missing rate (%)

1

2.4

1

2.4

1

2.4

1

2.4

1

2.4

11

26.8

6

14.6

Fig. 4

# A B

×10^3^ /µL

10

8

12

10

8

12

## Changes in platelet count

### (n = 14)

×10^3^ /µL

## Corrected changes in platelet count

### (n = 14)

day	day

6

4

2

0

6

4

2

0

120

100

80

60

40

20

0

1	2	3	4	5	6	7	8	9	10 11 12 13 14 15

120

100

80

60

40

20

0

1	2	3	4	5	6	7	8	9	10 11 12 13 14 15

The number of missing value

Missing rate (%)

3

21.4

1

7.1
