# Supplementary material for: Effect of linezolid on platelet count in critically ill patients with thrombocytopenia
Source: PLoS One. 2023 Jun 29;18(6):e0286088. doi: 10.1371/journal.pone.0286088 (PMC10310034; doi:10.1371/journal.pone.0286088)
Supplement: Attachment — Submitted filename: Reveiwer Comments with answer.docx [file pone.0286088.s004.docx]

| Editor Comments | Author Response |
| --- | --- |
| Abstract:  Line 19: remove TDM as it is not used again in the abstract  Line 19-21. Change to read: **monitoring and is readily available for critically ill patients. One adverse effect of LZD is pancytopenia, especially thrombocytopenia. We investigated the effect of LZD on platelet counts in critically ill patients, in the ICU, with thrombocytopenia**.  Line 23: after who add **had subsequently**    Line 26-32: Change to read: **The median (SE) platelet count prior to the initiation of LZD was 47 x10^3^ /uL** (please add SE). **This increased** **significantly to 86** **x10^3^ /ul on day 15 (p<0.01) with a median (IRQ) duration of LZD therapy of 9 days** (please add the IQR). **Thirty-two patients (58.2%) required PC transfusion in the 15 day study period. The daily rate of PC transfusion reduced from 30.2% on day 1-5 to 18.2% on day 11-15.**  Line 33-35: Change to read:  **Thrombocytopenia in critically ill patients in ICU did not worsen after the initiation of LZD therapy, and may be considered for the treatment of MRSA in this setting.** | Thank you for your detailed and careful review of our original article.  Removed as you suggested.  Changed as you suggested.  Added as you suggested.  Changed as you suggested.  Changed as you suggested. |
| Introduction:  Line 55: make Gram lower case  Line 56: Italicise enterococci  Line 61-62: Change the entire sentence, ‘On the other…effect of LZD’ to read:  One of the adverse effects of LZD is pancytopenia, especially thrombocytopenia  Line 66: after causes add **of** **thrombocytopenia,**  Line 68: add **critically ill** before patients and **in the intensive care unit (ICU) setting** after thrombocytopenia  Add a space between headings:  After line 68, 77, 105, 112, 139, 150 | Since this article focuses on LZD, which is effective against Gram-positive bacteria, we have decided to omit the description of Gram-negative bacteria.  Changed as you suggested.  Changed as you suggested.  Added as you suggested.  Added as you suggested.  Added as you suggested. |
| Study design:  Line 71: add the location and name of the hospital  Line 74-75: change ‘obtaining informed consent….patients’ to read, **informed consent was waived.**  Explain how the patients were made aware of the website, was this done verbally or was a hard copy of the details provided to the patients on discharge | Added as you suggested.  Changed as you suggested.  At our ICU, we provide patients and their families with an explanatory form stating that the clinical data may be used for future research and presentations. After that, when the research is approved, it is presented again on the website. |
| Patients and settings:  Line 79: after patients add, **in ICU**  Line 80: remove, **in our ICU**  Line 81: after count add, **of**  **Please add why the LZD was initiated in the patients, for treatment of MRSA?**  Line 82: what were the causes of death in those excluded. Please add if available.  Line 83: add the route of administration.  Line 84-85: change to read:  **of LZD discontinuation was decided by physicians and ICU staff at a daily ICR conference.**  Line 85-88: Change to read:  **The use of platelet concentrate (PC) transfusion was also decided at the daily ICU conference with indications including the platelet count, change in the platelet count, presence or absence of bleeding, treatment content such as blood purification therapy and necessity of treatment with high bleeding risk.**  Line 89: change that caused to, **and reasons for**  Line 90, 91: change ‘the first day to 15 days’ to read, **day 1 to 15**  Line 92-93: change to read. **The daily rate of PC transfusion (=PC transfused / all patients) was calculated over the 15 day study period and compared in three periods: early (days 1-5), middle period….**  Line 97: after on the, add, **first**  After when, add **the**  Line 99: change were to **was**  After compensated add **for**  After the average of add **the**  Line 102: add **(see Table 1).** | Changed as you suggested.  Added as you suggested.  Added the following sentence:  Sepsis patients admitted to the ICU often have AKI or undergo CRRT, and when selecting an anti-MRSA drug that requires dose adjustment, it takes time to adjust the blood concentration within the effective range. Therefore, we choose LZD for treatment of MRSA.  All death cases within 15 days after the start of LZD were due to acute or irreversible progression of the primary disease. The duration of LZD administration was 7 [6-8] days.  Inserted the following sentence:  Patients who died due to acute or irreversible progression of the primary disease within 15 days…  And, added the cause of death to Figure legends of Fig. 1.  Added “intravenously”as you suggested.  Changed as you suggested.  Changed as you suggested.  Changed as you suggested.  Changed as you suggested.  Changed as you suggested.  Added as you suggested.  Changed as you suggested.  Added as you suggested. |
| Statistical analysis: |  |
| Results:  It is not clear whether LZD was used for prophylaxis as well as treatment of the patients in this cohort. Please add this information in.  Line 114 and 115: change Patients to **Patient**  Line 115: Add the Table number i.e. Table 1  Line 131: Change sentence to read, **The changes in the mean platelet count for the original and corrected data are shown in Fig. 2A and 2B, respectively.**  Line 132-133: Change sentence, “Platelet count… ’ to read: **The mean (SE) platelet count was 47 ± 4x10^3^ /uL before LZD administration with a gradual but statistically significant increase over the 15 day period (p<0.01, for original and corrected data).**  Line 133, 143, 145, 156, 157: Before platelet add, **mean (SE)**  Line 135 and 147: than that before LZD administration’, change to, **than that before initiation of LZD** **therapy**  Line 135: It states that 32 patients required PC but in the table it mentions 27 patients ?  Line 136: change within to, **in the**  Line 137: add before early, **the**  Line 138 and line 160: remove ‘which was higher than’  Line 141: change ‘other than hematological’ to, **non-haematological**  Change ‘diseases were’ to, **diseases are**  Line 143: remove from the primary diseases.  Line 152: remove disease  Line 153: change ‘if’ to , **when**  Line 153: Change ‘While’ to, **The mean (SE)**  Line 159: change ‘within’ to, **in the** | Added the following sentence:  LZD was administered as empiric or definitive therapy.  Changed as you suggested.  Added as you suggested.  Changed as you suggested.  Changed as you suggested.  Added as you suggested.  Changed as you suggested.  The numbers in the Table 1 were wrong. Changed All to 32 in Table 1 and Non-hematological to 19 in Result and Table 1.  Changed as you suggested.  Added as you suggested.  Removed as you suggested.  Changed as you suggested.  Removed as you suggested.  Removed as you suggested.  Changed as you suggested.  Changed as you suggested.  Changed as you suggested. |
| **Discussion:**  **The structure of the discussion needs some adjustment:**  **The first section should deal with the thrombocytopenia (line 163-172, 187-. As you have, start by discussing all the literature which refers to LZD and thrombocytopenia line163-165, 194-195).**  **Then discuss your results, mention that the patients were critically ill patients in ICU and effectiveness of LZD in treating bacterial infections.**  **Discuss the reasons for the improvement in the platelet counts (line 195-199), how LZD induced thrombocytopenia may be masked by the improvement in platelet counts from other causes.**  **I would add that the data suggests that LZD is not implicated in the causing thrombocytopenia in this patient groups and could thus be considered for critically ill patients with thrombocytopenia due to infections or haematological diseases.**  **The section on renal failure and LZD should come after all the write-up on LZD and thrombocytopenia.**  **There are sections which contradict your statements, firstly you say that LZD is ideal as you don’t need to do TDM in RF and then shortly after you recommend that it should. Please reassess this section to read your thoughts clearly.**  **I would move the information referring to the pharmacokinetics of LZD to the introduction, speaking.**  **Put a space before the paragraph on limitations.**  **Line 211-213. Change the sentence ‘About the….anti-MRSA drugs’ to read, A single arm study design was chosen, as there was an insufficient number of patients who were receiving anti-MRSA drugs other than LZD, with which to compare data.**  **Line 213-215: Change the sentence ‘Secondly…next day’ to read, Secondly, there were missing platelet counts in several of the data sets, this was compensated for by calculating the average of measured values from the previous and subsequent days.**  **Line 215-217: Point three is not clear. Please reword to clarify.**  **Line 217-222: Reword ‘Fourthly….PC transfusion’ to read, Fourthly, it cannot be denied that PC transfusion resulted in a sustained increase in the platelet counts in some patients, however, since the frequency of PC transfusion progressively decreased from the early through to the late period, it was considered that the platelet count was recovering even under the administration of LZD.**  Line 222-223: Change the sentence ‘in other….general condition’ to read**, Additionally, LZD treated the underlying infection which was the cause of the initial thrombocytopenia in a proportion of the patients.**  Line 223-225: Change the sentence ‘Therefore, ….be ruled out.’ to, Therefore in certain patients, both the PC transfusions and LZD therapy may have led to the gradual increase in the platelet counts.  Line 225-227: Elaborate as to why this final comment is a limitation. It is not clear. | As you suggested, we rearranged the order of the discussion contents.  TDM of LZD is not required and dose adjustment based on renal function is not required generally in Japan. However, the relationship between renal function and the development of thrombocytopenia and the need for dose adjustment have reported. Changed to the following sentence:  The effect of renal function and CRRT on LZD concentration was not clear, in our cases. However, if TDM becomes common in Japan in the future, it may be better to adjust the dose of LZD in order to increase the safety and efficacy of LZD.  Moved “LZD has several features including…excellent tissue migration property.” to Introduction.  Putted a space.  Changed as you suggested.  Changed as you suggested.  It was hard to understand. Changed it to the following sentence:  Thirdly, the cause of thrombocytopenia before LZD administration was related to several factors, such as infection and administration drugs, and could not be clearly classified.  Changed as you suggested.  Changed as you suggested.  Changed as you suggested.  Since the part you pointed out is described in Patients and Methods, it was inappropriate to include it in the limitation. Removed as you suggested. |
| Conclusion:  Consider rewording:  **Thrombocytopenia in critically ill patients in ICU did not worsen after the initiation of LZD therapy, and may be considered for the treatment of MRSA in this setting. It would be advised that platelet counts are closely monitored with prompt discontinuation of LZD after resolution of the infection.**  I would add the final sentence on further studies line 234-235 to the discussion. | Changed as you suggested.  Added to the Discussion as you suggested. |
| Table1:  Add, the time period of the study and location to the title.  Change Age (year) to Age **(years)**  Median (IQR) to be added after:  N(%) to be added after:  Female  Male  For all the primary disease subcategories  For all the presumed cause of thrombocytopenia subcategories  And for all the categories on page 8  Female Haematological: change to 4 (28.6)  Male Haematological: change to 10 (71.4)  Non- haematological: change to 41 (74.5)  Haematological: change to 14 (25.5) | Added as you requested.  Changed as you suggested.  Added as you suggested.  Added as you suggested.  Changed as you suggested. |
| Figures:  2A and B, 3A and B, 4A and B.  Add a heading for the y axis i.e. platelet count  Expand the headings to include study date and location. | Added as you suggested.  Added as you requested. |
